# FAIM: Fairness-aware interpretable modeling for trustworthy machine learning in healthcare

**DOI:** 10.1016/j.patter.2024.101059

**Published:** 2024-09-12

**Authors:** Mingxuan Liu, Yilin Ning, Yuhe Ke, Yuqing Shang, Bibhas Chakraborty, Marcus Eng Hock Ong, Roger Vaughan, Nan Liu

**Affiliations:** 1 Centre for Quantitative Medicine, Duke-NUS Medical School, Singapore, Singapore; 2 Department of Anaesthesiology and Perioperative Medicine, Singapore General Hospital, Singapore, Singapore; 3 Programme in Health Services and Systems Research, Duke-NUS Medical School, Singapore, Singapore; 4 Department of Statistics and Data Science, National University of Singapore, Singapore, Singapore; 5 Department of Biostatistics and Bioinformatics, Duke University, Durham, NC, USA; 6 Department of Emergency Medicine, Singapore General Hospital, Singapore, Singapore; 7 Institute of Data Science, National University of Singapore, Singapore, Singapore

**Keywords:** AI fairness, fairness in healthcare, interpretable machine learning, trustworthy machine learning

## Abstract

The escalating integration of machine learning in high-stakes fields such as healthcare raises substantial concerns about model fairness. We propose an interpretable framework, fairness-aware interpretable modeling (FAIM), to improve model fairness without compromising performance, featuring an interactive interface to identify a “fairer” model from a set of high-performing models and promoting the integration of data-driven evidence and clinical expertise to enhance contextualized fairness. We demonstrate FAIM’s value in reducing intersectional biases arising from race and sex by predicting hospital admission with two real-world databases, the Medical Information Mart for Intensive Care IV Emergency Department (MIMIC-IV-ED) and the database collected from Singapore General Hospital Emergency Department (SGH-ED). For both datasets, FAIM models not only exhibit satisfactory discriminatory performance but also significantly mitigate biases as measured by well-established fairness metrics, outperforming commonly used bias mitigation methods. Our approach demonstrates the feasibility of improving fairness without sacrificing performance and provides a modeling mode that invites domain experts to engage, fostering a multidisciplinary effort toward tailored AI fairness.

## Highlights

• FAIM framework to tackle bias in healthcare
• FAIM improves model fairness while maintaining performance via near optimality
• FAIM features an interactive interface to enhance contextualized fairness
• FAIM links interpretability with fairness to verify the effects of bias mitigation

## The bigger picture

Fairness in clinical decision-making is a vital aspect of healthcare systems, and in the development of artificial intelligence (AI) algorithms, a lack of fairness can perpetuate existing biases and potentially harm patients. AI fairness aims to address and mitigate health disparities exacerbated by algorithmic decisions, promoting equitable healthcare delivery. However, the complex nature of clinical settings poses unique challenges, such as uncertainty in using non-clinical variables, addressing biases in clinical variables, and the severe consequences of wrong decisions. This complexity underscores the need for interpretability and domain expert involvement to contextualize AI fairness to specific clinical scenarios. The fairness-aware interpretable modeling (FAIM) framework assists in the selection of fairness-aware models in clinical settings while maintaining model performance.

## Graphical abstract

**Figure:**
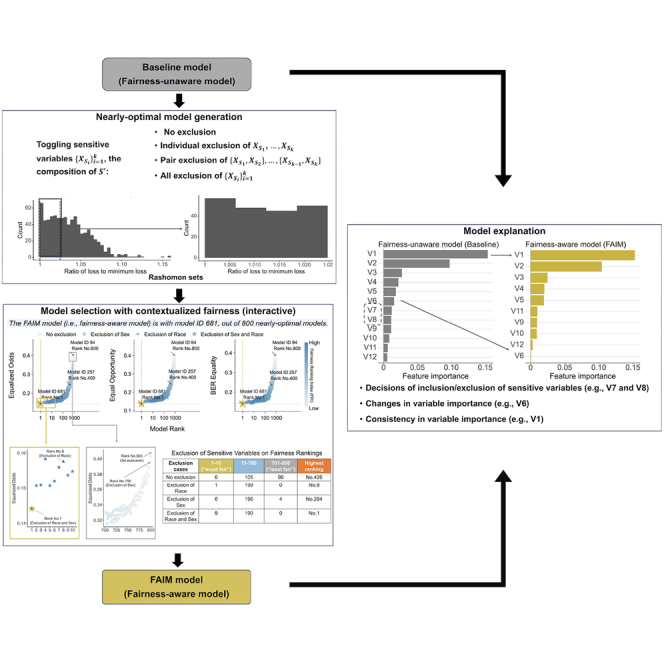

## Teaser

This study introduces FAIM, a fairness-aware and interpretable modeling framework designed to build well-performing yet fair models as alternatives to performance-optimized, fairness-unaware models. FAIM offers a new approach to bias mitigation by anchoring the performance of fairness-unaware models, exploring fairness diversity among nearly optimal models, and achieving contextualized fairness. The study shows that fairness can be improved without compromising performance, and by enhancing interpretability and fostering active engagement with domain experts, FAIM promotes contextualized AI fairness, encouraging clinician involvement and interdisciplinary collaboration.

## Introduction

As artificial intelligence (AI) gains prominence in high-stakes fields like healthcare, concerns about fairness have grown.^1^^,^^2^^,^^3^ Biases (as opposed to fairness) in machine learning arise when “sensitive” factors (e.g., age, sex, gender, race, ethnicity, socio-economic status, etc.) unjustly skew decision-making.^4^^,^^5^ In healthcare, a biased model can unjustly influence life-altering decisions, such as disease diagnosis^6^^,^^7^ and organ allocation.^8^ Clinical AI fairness is challenging due to healthcare’s complexity and the impact of social determinants, and its integration into clinical decision-making is crucial to prevent the escalation of health disparities.^9^^,^^10^^,^^11^

A seemingly straightforward approach to reduce bias is to simply exclude sensitive variables from the decision-making process, also named “under blindness,” but this has been deemed undesirable, particularly in cases where subpopulations (such as by sex) are distinct.^12^^,^^13^ Numerous methods have been developed to systematically mitigate biases, categorized by the stage of the modeling process at which they operate.^14^^,^^15^ Bias mitigation can occur pre-process through data adjustment (e.g., sampling^16^ and reweighing^16^), in-process via direct fair model development (e.g., regularization^17^^,^^18^^,^^19^ and representation learning^20^^,^^21^), or post-process by altering model outputs (e.g., equalized-odds post-process^22^). However, pre-process methods often encounter challenges when addressing biases that involve intersecting multiple attributes (e.g., race and sex).^23^^,^^24^ Post-process approaches, by modifying outputs, fail to address the root causes of biases, leaving predictions altered but not clarified.^25^

Fairness in machine learning is hindered by a lack of interpretability, especially those employing models with black-box architectures for bias mitigation. Interpretability concerns also arise when randomness is utilized to post-process individual predictions (e.g., changing a positive prediction into a negative one) for the purpose of group fairness without clear clinical justification.^22^^,^^26^^,^^27^ In healthcare, the contextual nature of fairness necessitates the specialized knowledge of clinicians to ensure that fairness definitions align with clinical realities, as AI fairness and its decision-making consequences differ markedly across medical fields.^11^^,^^12^ However, the lack of interpretability poses formidable obstacles to their active participation in the modeling process, challenging the mutual understanding between AI developers and clinicians and impeding the translation of technical advancements into clinical practice.

Another common limitation of existing bias mitigation methods is that they often compromise model performance, as measured by machine learning metrics like area under the receiver operating characteristic (ROC) curve (AUC). This drawback can hamper practical application, cast doubt on the claimed fairness due to increased uncertainty, and potentially lead to severe and unforeseen consequences. According to a recent empirical study,^28^ current bias mitigation methods degraded model performance in approximately 50% cases, whereas in 25% cases, they worsened both fairness and performance. Specifically, Pfohl et al.^29^ empirically demonstrated that the methods penalizing prediction discrepancies (which are often interpreted as evidence of bias) can almost universally decrease multiple aspects of performance. Nevertheless, it has been empirically shown that such a trade-off between model performance and fairness is not inevitable.^5^^,^^27^

**Figure 1:**
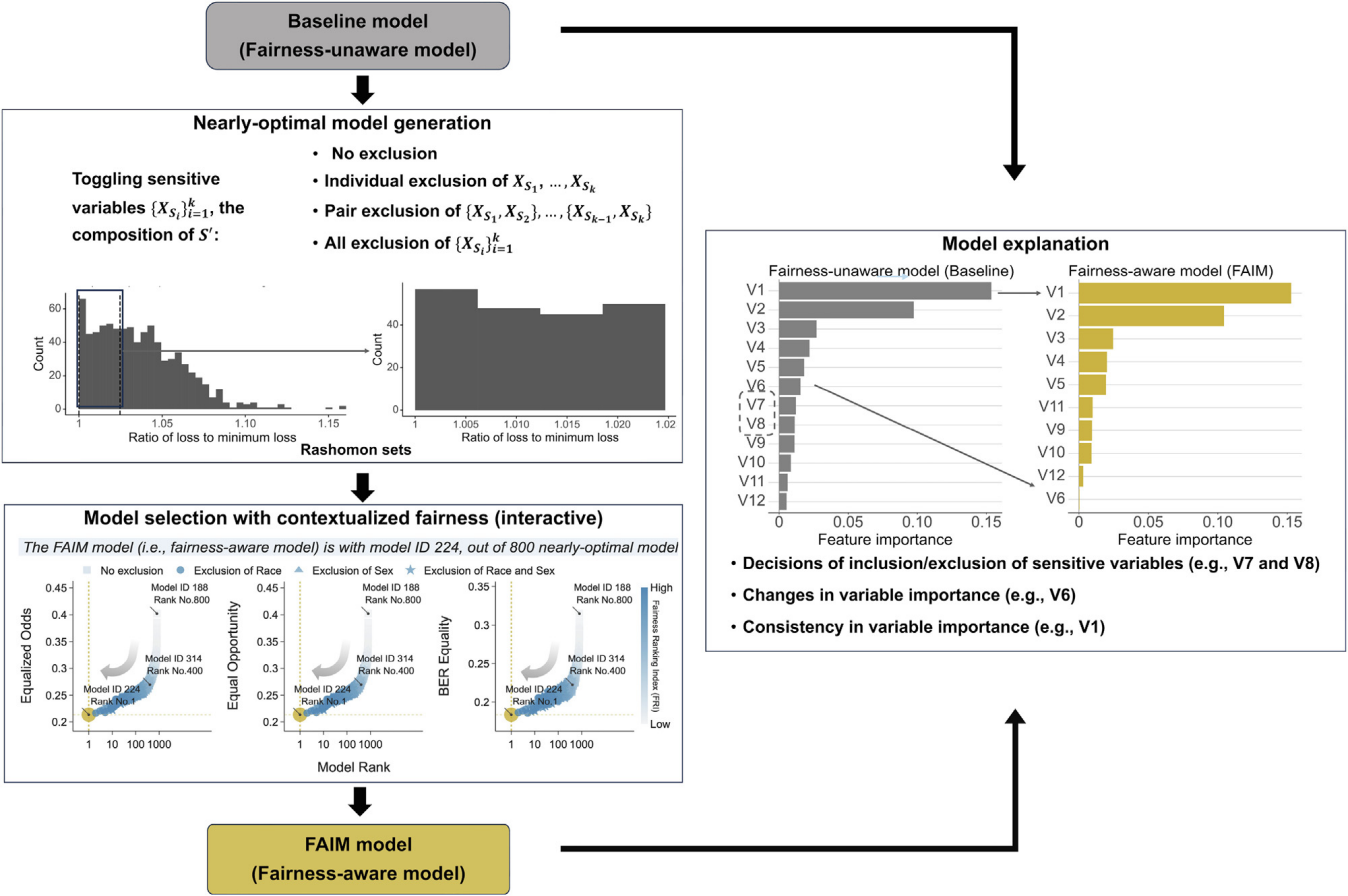
The general framework of FAIM The FAIM framework consists of three modules to locate a fairer model in a set of nearly optimal models: nearly optimal model generation, model selection with contextualized fairness, and model explanation.

To bridge the gap in clinical AI fairness, we propose a new fairness-aware interpretable modeling (FAIM) framework (Figure 1) to achieve fairness with enhanced interpretability without sacrificing model performance. FAIM operates within a set of nearly optimal models that offer high performance without necessarily reaching optimality.^30^ By leveraging the varying degrees of model reliance on variables (including sensitive variables) within the set,^31^^,^^32^ FAIM can identify alternative model formulations that improve model fairness without significantly impairing performance. Rather than producing a single final model and leaving uncertainty about which aspects of fairness are compromised for performance, FAIM utilizes models’ diverse fairness profiles and near-optimal performance to facilitate informed discussions between clinicians and model developers to prioritize fair models for specific contexts. FAIM also examines the impact of excluding some (or all) sensitive variables on model fairness, visualizes the findings to help clinicians select a fairness-enhanced model with reasonable interpretation and contextualization, and employs explainable AI (XAI) methods to clarify variable importance changes due to the fairness enhancement to further improve interpretation. We illustrate our method in the prediction of hospital admission in the emergency department (ED) using two large-scale clinical datasets, focusing on reducing potential bias due to two sensitive variables, i.e., race and sex. Although we use clinical case studies, the framework introduced has broader applicability across multiple domains and diverse tasks.

## Results

We implemented FAIM on two large clinical datasets to predict hospital admission: the Medical Information Mart for Intensive Care IV ED (MIMIC-IV-ED)^33^ and data collected from the Singapore General Hospital ED (SGH-ED),^34^ with the characteristics summarized in Tables S1 and S2. We aimed to mitigate biases related to sex and race, denoted by $X_{s}$, and other variables were considered less sensitive, denoted as $X_{U}$, due to their uncertain roles regarding race and sex biases. Particularly, the variable of age was considered less sensitive because of its known high predictive ability for hospital admission. We used logistic regression as the fairness-unaware baseline model ("baseline” for short), aligning with clinical studies that value not only the discrimination performance but also model transparency and statistical functionality (e.g., odds ratios for predictors).^35^ As demonstrated in the two datasets, the FAIM framework transparently navigated the exclusion of sensitive variables, and the FAIM output models (i.e., fairness-aware models, “FAIM” for short) had satisfactory discriminatory performance and significantly improved model fairness compared to other commonly used bias mitigation methods.

### Model selection among nearly optimal models

**Figure 2:**
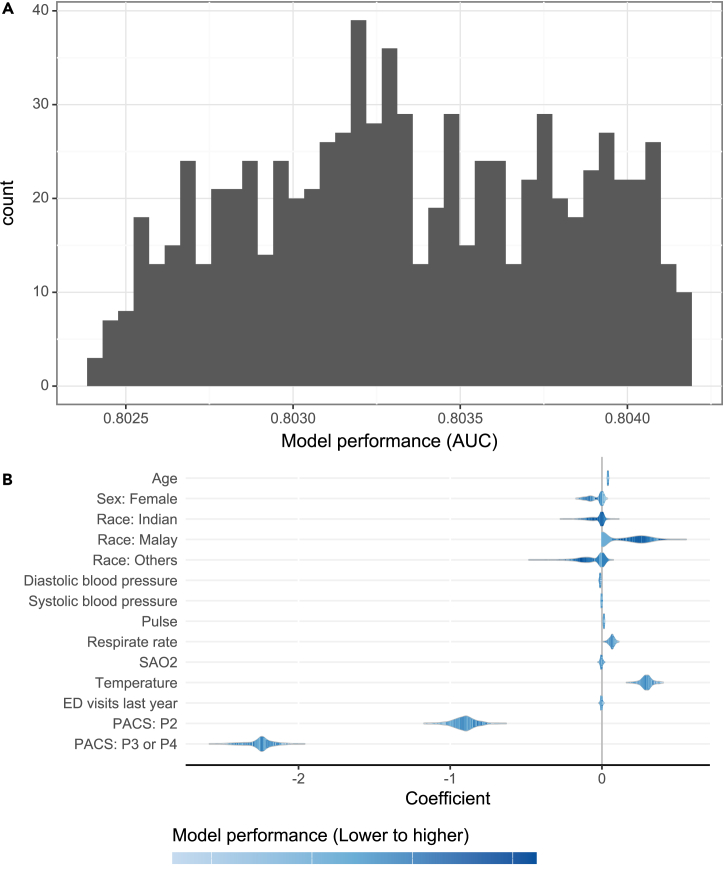
Distribution of model performance and coefficient variability for nearly optimal models (*n* = 800) on SGH-ED data (A) Histogram showing the distribution of model performance (AUC) for nearly optimal models evaluated on the validation set. (B) Violin plot depicting the variability of model coefficients across different variables. The width of each violin represents the range of coefficient values, while the color gradient indicates the level of model performance, with darker shades corresponding to higher performance. The variables PACS triage score, temperature, race, and sex display wide spectra of coefficients.

**Figure 3:**
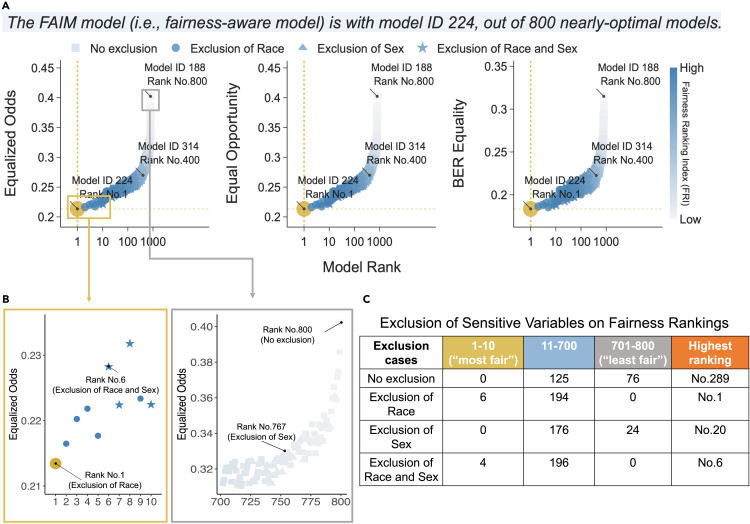
The interactive plot for model selection based on the nearly optimal models’ fairness evaluated on the validation set of SGH-ED data (A) The graphical interface ranks nearly optimal models based on fairness metrics—equalized odds, equal opportunity, and BER equality. Model 224 is ranked first by the fairness ranking index (FRI), highlighted as the default fairness-aware model. Users can interactively engage with the data by hovering over points to display model details, zooming in on sections like the top 10 models, and adjusting panel views. (B) Illustration of the panel of equalized odds metric with top 10 models showcased in the golden box, showing four models (stars) excluding both sex and race and six models (circles) excluding only race. The bottom 100 models, showcased in the gray box, predominantly include no exclusions (rectangles), with a minority excluding sex (upward triangles). (C) A tabulation of models based on the exclusion cases of sensitive variable(s) detailing the counts of models in different fairness ranges—from “most fair” to “least fair.” The rightmost column records the highest ranking obtained by the nearly optimal models for each exclusion case.

After generating a set of nearly optimal models (see details in subsection nearly optimal model generation in the experimental procedures), we located the fairness-aware model via the interactive interface of FAIM. For the SGH-ED data, Figure 2 shows the distribution of coefficients for the nearly optimal models, showcasing various spectra of coefficients for sensitive variables sex and race as well as less-sensitive variables such as temperature and triage score (i.e., patient acuity category scale [PACS] used in Singapore). Such variation in coefficients suggests that while model performance is not impacted (Figure 2A), these nearly optimal models respond differently to variables, providing room for fairness improvement. Figure 3 showcases the fairness distribution of these nearly optimal models, aiding in model selection with data-driven evidence. In Figure 3A, regarding individual fairness metrics (equalized odds, equal opportunity, and balanced error rate [BER], as defined in Table 1), these nearly optimal models displayed diverse fairness profiles. Across panels, these models were jointly assessed and ranked using the proposed fairness ranking index (FRI), which aggregates these individual fairness metrics for a comprehensive assessment of fairness (see the experimental procedures for a detailed definition). As visualized in Figure 3B and quantified in Figure 3C, among the top 10 models, the top 5 models excluded race, whereas the sixth-ranked model excluded both race and sex (shown in the golden box); conversely, none of the models in the bottom 100 excluded race (shown in the gray box), which suggested the necessity of excluding race. We selected the top 1 model (i.e., model ID 224) as the FAIM output, which included the sex variable. This decision was informed by the similar distribution of nearly optimal models regardless of sex variable exclusion, indicating minimal bias caused by sex (Figure 3C), coupled with the absence of clinically detected sex biases in Singapore’s hospital admissions so far.

**Table 1:** Separation-based fairness metrics: Formulas and descriptions

Fairness metrics^a^	Formulas	Description
Equalized odds	$\max\left\{\underset{S}{Range}\left(P\left[\hat{Y}=1|Y=1,X_{S}\right]\right),\underset{S}{Range}\left(P\left[\hat{Y}=1|Y=0,X_{S}\right]\right)\right\}$	this metric checks if both true positive rates and false positive rates are consistent among subgroups, indicating that the likelihood of a correct or incorrect prediction does not vary significantly among subgroups
Equalized opportunity	$\underset{S}{Range}\left(P\left[\hat{Y}=1|Y=1,X_{S}\right]\right)$	this metric assesses if the true positive rates (sensitivity) are consistent among subgroups, ensuring that the model’s ability to correctly identify positive cases is consistent among subgroups
Balanced error rate equality	$\underset{S}{Range}\left(P\left[\hat{Y}=1|Y=0,X_{S}\right]+P\left[\hat{Y}=0|Y=1,X_{S}\right]\right)$	this metric evaluates if the balanced error rate, which is the sum of the false positive rate and the false negative rate, is similar across subgroups, indicating balanced misclassification rates among subgroups

a These three metrics are limited to binary outcomes. The smaller values indicate better fairness and fewer biases.

For the MIMIC-IV-ED dataset, Figure S1 shows the various spectra of coefficients for sensitive variables and less-sensitive variables, such as temperature and triage score (i.e., emergency severity index [ESI] used in the US). FAIM’s default choice was the top 1 model (i.e., model ID 681) with both race and sex excluded. This preference aligned with nine out of the top 10 models, as depicted in Figure S2. Similarly, we retained the top 1 model as the FAIM output model, reflecting the majority preference of the top-ranking models and addressing known racial and sex disparities in ED care outcomes in the US.^36^^,^^37^^,^^38^

### Evaluation of model fairness

With the FAIM models above, we evaluated model fairness on the split-out test set. FAIM consistently outperformed both baseline models (i.e., fairness-unaware models) and other bias mitigation methods in model fairness except for the reductions method (see Table 2 for MIMIC-IV-ED data and Table 3 for SGH-ED data). FAIM’s improvement in fairness was statistically significant (*p* < 0.001), yielding a 53.5%–57.6% enhancement in fairness metrics for the MIMIC-IV-ED case and a 17.7%–21.7% enhancement for the SGH-ED case, compared with baseline models. Importantly, while the reductions method did improve fairness, it came at the cost of prediction performance, which led to impartially poor performance across different subgroups (more details in the next subsection, evaluation of model performance and statistical functionality).

**Table 2:** Two-dimension evaluation of model fairness and performance for bias mitigation methods using MIMIC-IV-ED data

	Fairness metrics^a^			Performance metrics		
	Equal opportunity	Equalized odds	BER equality^b^	AUC	Sensitivity^c^	Specificity^c^
Baseline^d^	0.316	0.316	0.301	0.790 [0.787, 0.793]	0.715 [0.711, 0.720]	0.724 [0.720, 0.728]
Under blindness^e^	0.159	0.159	0.155	0.787 [0.784, 0.790]	0.707 [0.703, 0.711]	0.729 [0.724, 0.733]
FAIM	0.134	0.147	0.140	0.786 [0.783, 0.789]	0.725 [0.720, 0.729]	0.711 [0.707, 0.715]
Reweighing (pre-process)	0.206	0.206	0.183	0.789 [0.786, 0.792]	0.709 [0.704, 0.713]	0.731 [0.726, 0.735]
Reductions (in-process)	0.037	0.037	0.032	0.706 [0.703, 0.709]	0.657 [0.652, 0.662]	0.755 [0.751, 0.759]
Fair-GLM (in-process)	0.146	0.151	0.148	0.786 [0.783, 0.789]	0.719 [0.714, 0.723]	0.715 [0.711, 0.720]
Equalized-odds post-processing (post-process)	0.734	0.734	0.554	0.594 [0.591, 0.596]	0.335 [0.330, 0.340]	0.852 [0.849, 0.855]
Adnet^f^ (in process)	0.158	0.189	0.174	0.743 [0.740, 0.747]	0.703 [0.699, 0.708]	0.660 [0.656, 0.664]

a Smaller values indicate higher levels of fairness.

b BER: equality of the combination of the true positive rate and the true negative rate.

c The thresholds were determined by Youden’s J index for methods that can yield predictive probabilities (i.e., original logistics regression, FAIM, reweighing, Fair-GLM, Adnet, and under blindness). The in-process method reductions and the post-process method equalized-odds post-process directly generated the binary prediction.

d Baseline: the original logistics regression model, i.e., fairness-unaware baseline model.

e Under blindness: the logistics regression with sensitive variables excluded.

f Adnet: bias mitigation method utilizing adversarial learning, i.e., black-box models, instead of manipulating regression models to achieve fairness.

**Table 3:** Two-dimension evaluation of model fairness and performance for bias mitigation methods using SGH-ED data

	Fairness metrics^a^			Performance metrics		
	Equal opportunity	Equalized odds	BER equality^b^	AUC	Sensitivity^c^	Specificity^c^
Baseline^d^	0.299	0.299	0.237	0.804 [0.803, 0.806]	0.713 [0.710, 0.715]	0.753 [0.751, 0.755]
Under blindness^e^	0.252	0.252	0.209	0.803 [0.802, 0.805]	0.720 [0.717, 0.722]	0.744 [0.742, 0.746]
FAIM	0.234	0.234	0.195	0.802 [0.801, 0.804]	0.719 [0.717, 0.721]	0.746 [0.744, 0.748]
Reweighing (pre-process)	0.244	0.244	0.212	0.803 [0.802, 0.805]	0.712 [0.710, 0.715]	0.750 [0.748, 0.752]
Reductions (in-process)	0.035	0.036	0.036	0.664 [0.662, 0.665]	0.542 [0.540, 0.545]	0.785 [0.784, 0.787]
Fair-GLM (in-process)	0.260	0.260	0.216	0.802 [0.801, 0.804]	0.716 [0.713, 0.718]	0.747 [0.745, 0.748]
Equalized-odds post-processing (post-process)	0.685	0.685	0.490	0.601 [0.600, 0.603]	0.327 [0.323, 0.330]	0.875 [0.874, 0.876]
Adnet^f^ (in-process)	0.357	0.357	0.337	0.748 [0.747, 0.750]	0.695 [0.693, 0.698]	0.675 [0.673, 0.677]

a Smaller values indicate higher levels of fairness.

b BER equality: equality of the combination of the true positive rate and the true negative rate.

c The thresholds were determined by Youden’s J index for methods that can yield predictive probabilities (i.e., original logistics regression, FAIM, reweighing, Fair-GLM, Adnet, and under blindness). The in-process method reductions and the post-process method equalized-odds post-process directly generated the binary prediction.

d Baseline: the original logistics regression model, i.e., fairness-unaware baseline model.

e Under blindness: the logistics regression with sensitive variables excluded.

f Adnet: bias mitigation method utilizing adversarial learning, i.e., black-box models, instead of manipulating regression models to achieve fairness.

Our ablation study suggests that FAIM’s advantages extend well beyond merely omitting sensitive variables, referred to as the under-blindness method. Indeed, regarding fairness metrics, FAIM’s performance improvement over under blindness was notable—ranging from 7.55% to 15.72% for the MIMIC-IV-ED dataset (Table 2) and from 6.69% to 7.14% for the SGH-ED dataset (Table 3).

Beyond fairness metrics, we conducted subgroup analyses by sensitive variables to evaluate FAIM’s impact on fairness. Table 4 shows that FAIM substantially reduced disparities in both true positive and true negative rates across racial subgroups for both datasets. For sex subgroups, FAIM notably reduced the disparities for the MIMIC-IV-ED dataset, while its effect on the SGH-ED dataset was not consistently significant, given the relatively small initial disparities present in the baseline model.

**Table 4:** Subgroup analysis regarding sex and race

		MIMIC-IV-ED			SGH-ED		
		Baseline	Under blindness^a^	FAIM	Baseline	Under blindness	FAIM
Race	ΔTPR^b^	0.251	0.133	0.122^c^	0.264	0.223	0.211^c^
Race	ΔTNR^d^	0.224	0.130	0.129^c^	0.170	0.149	0.134^c^
Sex	ΔTPR	0.074	0.025	0.018^c^	0.015	0.001^c^	0.005
Sex	ΔTNR	0.086	0.034	0.027^c^	0.008	0.025^c^	0.027

a Under blindness: the logistics regression with sensitive variables excluded.

b ΔTPR: the gap of true positive rate among race/ethnicity or sex subgroups.

c Method that yields the least disparity.

d ΔTNR: the gap of true negative rate among race/ethnicity or sex subgroups.

**Figure 4:**
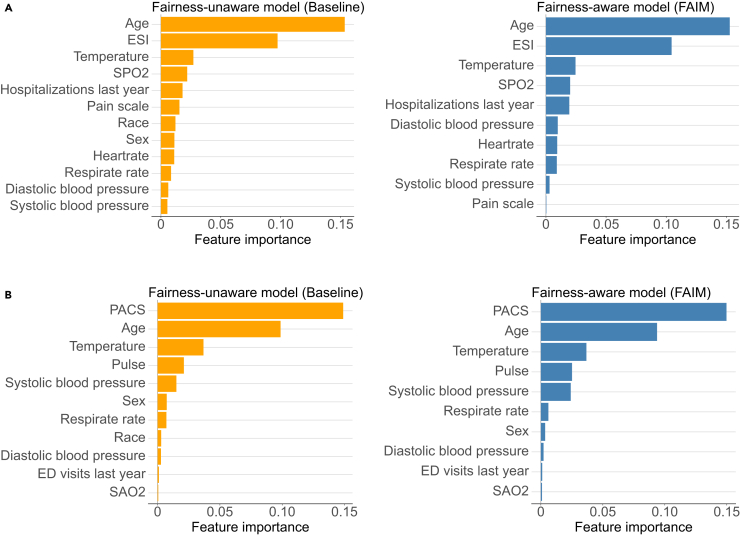
Variable importance analysis in both fairness-unaware (i.e., baseline) and fairness-aware (i.e., FAIM) models (A) Results based on MIMIC-IV-ED dataset. (B) Results based on SGH-ED dataset. The fairness-aware model refers to the ranked no. 1 fairest model yielded by FAIM, and the fairness-unaware model refers to the baseline model, i.e., the logistics regression model.

To further explore the changes in model reliance on the variables that lead to the improvement in fairness by FAIM, we analyzed this by comparing the variable importance between the fairness-unaware (i.e., baseline) and fairness-aware (i.e., FAIM) models, measured by Shapley additive explanations (SHAP).^39^ Beyond the sensitive variables of sex and race, for both datasets, the FAIM models preserved the variable contributions of less-sensitive variables, similar to the baseline models, while making minor changes to address fairness. As indicated by SHAP (Figure 4), the FAIM models preserved the importance of triage scores—ESI for MIMIC-IV-ED and PACS for SGH-ED—but downplayed the “pain scale” in the MIMIC-IV-ED case (Figure 4A).

### Evaluation of model performance and statistical functionality

With improved model fairness, we then evaluated FAIM from the perspective of model performance. Our analysis of classification metrics—AUC, sensitivity, and specificity, detailed in Tables 2 and 3—revealed that FAIM maintained a comparable performance to the baseline models. For the MIMIC-IV-ED dataset, FAIM achieved an AUC of 0.786 (95% confidence interval [CI]: 0.783–0.789), which closely aligned with the baseline model’s AUC of 0.790 (95% CI: 0.787–0.793). Similarly, in the SGH-ED dataset, FAIM’s AUC was 0.802 (95% CI: 0.801–0.804), which closely matched the baseline model’s AUC of 0.804 (95% CI: 0.803–0.806).

The sensitivity and specificity values achieved by FAIM were also on par with the baseline models. In the MIMIC-IV-ED data, FAIM achieved a sensitivity of 0.725 (95% CI: 0.720–0.729) and a specificity of 0.711 (95% CI: 0.707–0.715), compared to the baseline model with a sensitivity of 0.715 (95% CI: 0.711–0.720) and a specificity of 0.724 (95% CI: 0.720–0.728). In the SGH-ED data, FAIM delivered a sensitivity of 0.719 (95% CI: 0.717–0.721) and a specificity of 0.746 (95% CI: 0.744–0.748), while the baseline model had a sensitivity of 0.713 (95% CI: 0.710–0.715) and a specificity of 0.753 (95% CI: 0.751–0.755). Among other bias mitigation methods, in-process methods such as reductions and post-process methods such as equalized-odds post-process heavily degraded the classification performance.

**Figure 5:**
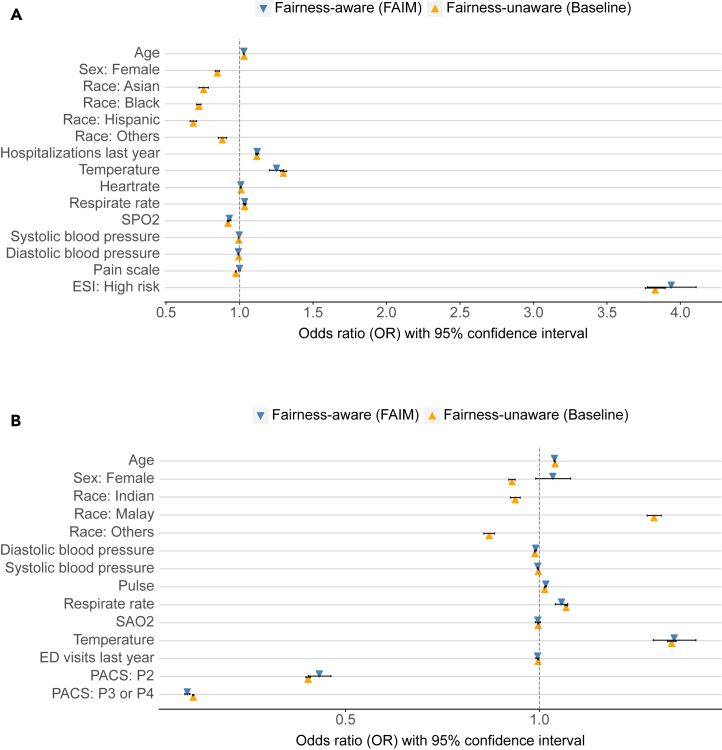
Comparison of the odds ratios between fairness-unaware (i.e., baseline) and fairness-aware (i.e., FAIM) models (A) Results based on SGH-ED dataset. (B) Results based on MIMIC-IV-ED dataset. The framework of FAIM excluded both sensitive variables race and sex from modeling to address fairness. As a result, the FAIM model did not have odds ratios for these two variables. Similarly, on the SGH-ED dataset, the variable race was excluded.

FAIM models’ odds ratios, as well as the corresponding statistical significance, were closely aligned with the fairness-unaware baseline models, with minor changes addressing fairness (Figure 5). This contrasts with some conventional bias mitigation methods, especially those based on black-box models, which often compromise statistical functionality. The sensitive variables race and/or sex, which were statistically significant in the baseline models, were automatically excluded owing to their minimal effects on model predictive ability. Specifically, in the case of MIMIC-IV-ED data, the pain scale variable became less significant in FAIM (Figure 5), aligning with its reduced variable importance as shown in SHAP analyses (Figure 4).

In terms of runtime efficiency, FAIM requires moderate training time—more than simpler methods like reweighing and under blindness, comparable to regularization-based methods and less than representation-learning-based methods due to the absence of hyperparameter tuning (see Table S3).

## Discussion

The pursuit of AI fairness is of increasing importance, particularly in high-stakes decision-making.^1^^,^^2^^,^^3^ We contribute to this field by introducing FAIM, a fairness-aware, interpretable framework, to build well-performing yet fair models that can serve as alternatives to the fairness-unaware model that is solely optimized for performance. We provide a different mindset for bias mitigation, that is, to anchor the performance achieved by the fairness-unaware model, explore the diversity of fairness among the nearly optimal models, and achieve contextualized fairness. Our findings reveal that fairness can be enhanced without sacrificing model performance. By enhancing interpretability and fostering active engagement with domain experts based on a diverse set of nearly optimal models, FAIM promotes contextualized AI fairness, encouraging clinician involvement and multidisciplinary collaboration.

### Enhancing model fairness without impairing model performance

Beyond merely enhancing fairness, it is crucial for bias mitigation methods to uphold satisfactory performance (e.g., AUC). A model’s performance is paramount; without it, the model’s utility in clinical decision-making is compromised.^40^ Some fairness methods, particularly post-process methods, often sacrifice model performance,^41^ raising concerns about their practicality. Our evaluations also reveal that these methods tend to reduce overall AUCs without reliably diminishing disparities (for example, equalized-odds post-process, as shown in Tables 2 and 3). This reduction in predictive performance is likely due to the randomness introduced in post-processing to achieve a specific fairness metric (e.g., equalized odds), which becomes even less effective with more subgroups, considering the intersectional effects of sensitive variables crucial in clinical settings.^22^^,^^23^ Pre-process methods such as reweighing also face challenges with an increasing number of subgroups, as they assume equal positive rates among all subgroups.^16^ Regularization-based methods may skew the optimization objective toward the majority class,^25^ while adversarial-learning-based methods can be unstable during training and sensitive to hyperparameters.^42^ Furthermore, bias mitigation methods often view fairness and performance as conflicting objectives, typically constraining one to optimize the other,^17^^,^^18^^,^^19^^,^^20^^,^^21^ focusing on the trade-off between performance and fairness and actually leaving a single model candidate at a fixed performance level. FAIM questions whether a single model can directly and accurately capture the fairness in each specific context and addresses that at a performance level, the range of model fairness can be wide, and these models may differ in coefficients or parameters (Figures 2 and S1). Anchoring the performance achieved by the model solely optimized for performance, FAIM investigates the diversity of model reliance on variables and its impact on fairness.

Statistical functionality, for example, odds ratios and their CIs, is also essential for elucidating model outputs and informing decision-making, as is commonly needed in clinical studies. Hence, it may be insufficient to merely provide binary predictions without details about why a model made a certain prediction or under what conditions the predictions hold, as is the case with most conventional bias mitigation methods. In the demonstration with the logistic regression model on both MIMIC-IV-ED and SGH-ED datasets, FAIM could output similar odds ratios and CIs for most less-sensitive variables, aligning with baseline models, with some adjustments to address fairness (Figure 5). These findings indicate that FAIM can provide a viable and fairness-aware alternative to the original machine learning models that are optimized for performance.

### The importance of interpretability in fairness modeling

The interpretability in FAIM is multilayered. Firstly, its underlying principle is intuitive, which is to prioritize fairer models among a set of nearly optimal models to improve fairness. The model family to which the fairness-unaware model as well as all the nearly optimal models belong is inherently interpretable. In addition, the process of model selection is transparent and interactive, as shown in Figure 3 (described in the subsection model selection with contextualized fairness in the experimental procedures). Such an interactive process can assist in integrating data-driven evidence and clinical expertise, especially regarding sensitive variables that require contextualized consideration rather than a one-fits-all approach. Moreover, the variable importance analyses (e.g., using SHAP in our examples) can illustrate changes in variable contribution between fairness-unaware and fairness-aware models, for example, the diminished importance of the variable “pain description” on MIMIC-IV-ED data.

While many methods may produce bias-mitigated predictions, they may not always fundamentally address algorithmic bias or elucidate the modifications made to enhance fairness.^25^ For example, the post-process method FaiRS developed by Coston et al.^43^ is also grounded in near optimality. FaiRS thoughtfully adjusts binary predictions to promote group fairness, occasionally by altering a negative prediction to a positive one under the selective labels setting. FAIM further underscores the role of model interpretability in improving fairness and showcases that model interpretability can serve as a tool to disclose model information, verify the effects of bias mitigation strategies, and potentially enhance trust.

### Keeping domain experts in the loop

Clinical evidence plays a crucial role in reducing model biases, especially when dealing with sensitive variables in the decision-making process. The results of FAIM in the MIMIC-IV-ED case resonate with literature that highlights racial and sex disparities in ED care outcomes in the US.^36^^,^^37^^,^^38^ Beyond excluding sensitive variables, FAIM also attenuated the variable of pain description, which may carry inherent sex biases.^44^^,^^45^

Model fairness can have geographical variations.^23^ As detailed in Table 4, the original models displayed race and sex disparities that were more pronounced in the US dataset compared to the Singapore dataset, and FAIM also showed varying effects in addressing these disparities. Additionally, FAIM addresses disparities across both race and sex in the US data while addressing primarily race in the Singapore data.

Advancing AI fairness in healthcare necessitates domain expertise to steer the process of bias mitigation effectively. FAIM generates and operates within a set of nearly optimal models that maintain performance comparable to the optimal model while exhibiting varying degrees of variable reliance (e.g., temperature), leading to various fairness profiles (Figures 2, 3, S1, and S2). Such diversity in fairness within the near optimality of performance allows for meaningful interactions between domain experts and algorithm developers to address specific fairness issues within the clinical context. Particularly, FAIM’s user-friendly graphical interface enables clinicians to influence model development with their insights directly. It also helps the validation of clinical judgment with data-driven evidence and encourages dialogue in the presence of divergences. For example, if including sensitive variables improves fairness, then this may suggest a need to investigate systematic biases or verify corresponding biological differences across subgroups, which can be embedded within involved less-sensitive variables. Sensitive variables may act as proxies for model adjustment, as in association studies, to better estimate the treatment effects. However, in prediction models, including such variables can directly influence predicted risk and associated actions, potentially raising ethical concerns. Through FAIM, clinicians can verify and reinforce this ethical consideration, potentially leading to more informed and ethically sound model adjustments. These insights on fairness notions, critical metrics, and clinical evidence related to sensitive variables can ultimately shape a more equitable and contextualized approach to patient care.

### The scalability of FAIM and future works

FAIM advocates that bias mitigation should occur concurrently with the modeling process, instead of as a one-time step, given that all stages can inject bias into the final decision-making.^11^ Despite FAIM being an in-process-type method, it can synergize with pre-process strategies like reweighing to tackle data underrepresentation, which is a known source of data bias, particularly when subgroup patterns are pronounced.^41^ Additionally, methods that uncover data biases and abnormalities^46^^,^^47^ can be integrated into the framework of FAIM for data pre-processing.

In addition to its flexibility in module plug in, the framework of FAIM also offers adaptability to other machine learning models (e.g., neural networks) in the future. Such adaption requires additional work for different model types.^31^ The sampling distribution of coefficients may not always be applicable; however, it could be executed via empirical procedures, such as generating random masks for weight matrices for neural networks, or model-specific theoretical techniques, such as dynamic programming methods to enumerate the trees for decision trees.^31^ Catering to the model types, other explanation methods (e.g., Grad-CAM^48^ for deep learning models) can be used beyond SHAP to more accurately explain model behaviors to investigate the model changes when addressing fairness.

Although our study focused on separation-based metrics like equalized odds, equal opportunity, and BER equality, which are widely used in practice and relate directly to essential model performance, the FAIM framework can also integrate other widely used fairness definitions, for example, independence-based metrics^14^ such as statistical parity into the FRI composition. Independence-based metrics are useful when the outcome of interest lacks a reliable ground truth, rendering separation-based metrics unreliable.^49^ However, caution is advised in using independence-based metrics given the risk of misapplication, particularly in complex contexts of healthcare where biological differences and social biases often intertwine with each other.^11^^,^^49^^,^^50^ Moreover, despite current fairness metrics predominantly addressing binary outcomes,^14^ extending FAIM to handle other outcome types (e.g., time-to-event outcomes^51^) with customized fairness metrics is a promising direction for future work.

While FAIM was designed with a focus on clinical AI fairness, its versatility extends to other high-stakes domains, such as finance and criminal justice. See Note S1 for the summarized results using the COMPAS data,^52^ showing the benefits of FAIM when forcing the exclusion of sensitive variables from modeling. Notably, achieving AI fairness can present inherently distinct challenges across fields. The involvement of domain experts in the modeling process is indispensable, as their deep expertise can ensure that models are grounded in the nuanced realities of each domain. This further emphasizes the crucial role of keeping humans in the loop to navigate the complexities effectively.

### Limitations

This study has several limitations. Firstly, we only demonstrated our method in two datasets related to emergency medicine. Further validation is needed in a wide spectrum of clinical applications. Additionally, the procedure of pinpointing nearly optimal models may benefit from further optimization. Lastly, there is room for improvement in the manner of obtaining CIs for the alternative odds ratios to more closely align with the functionality of the original models.

### Conclusion

FAIM is an interpretable and interactive framework capable of generating fairness-aware models. These models yielded by FAIM can achieve a significant improvement in fairness without compromising the performance and functionality. The interpretability of this approach invites domain experts into the heart of the modeling process, fostering a richer multidisciplinary collaboration. This collaboration is crucial for crafting AI fairness to specific contexts, thereby aiding in fair and just clinical decision-making and ultimately advancing equitable AI in healthcare.

## Experimental procedures

### The FAIM framework

The FAIM framework consists of three modules to locate a “fairer” model in a set of nearly optimal models: nearly optimal model generation, model selection with contextualized fairness, and model explanation, as visualized in Figure 1. Let *Y* denote the outcome, $\hat{Y}$ denote the predictions, $X_{S}$ = $\left(X_{s_{1}},\ldots ,X_{s_{k}}\right)$ denote a set of k sensitive variables (e.g., sex and race), $X_{S^{\prime }}$ represent a feature subset of sensitive variables in $X_{S}$, and $X_{U}=\left(X_{U_{1}},\ldots ,X_{U_{p}}\right)$ collectively denote p less-sensitive variables. Notably, $X_{U}$ are variables that do not directly contain sensitive information and cause bias, but as shown in the clinical examples, some seemingly non-sensitive variables may indirectly link to sensitive information and be affected after fairness enhancement. A model built with variables $X_{U}$ and $X_{S^{\prime }}$ is denoted by $f\left(X_{U},X_{S^{\prime }}\right)$. The loss function of this model is denoted as $L\left(f\left(X_{U},X_{S^{\prime }}\right),Y\right)$, along with the expected loss $E\left[L\left(f\left(X_{U},X_{S^{\prime }}\right),Y\right)\right]$.

### Nearly optimal model generation: Composing IRSs with multiple sensitive variables

Conventional efforts to develop an optimal model typically focus on model performance, which is to fit the data and minimize the expected loss functions. Nevertheless, as one approaches the theoretically optimal model due to the data uncertainty, many models can fit the data with comparable loss, forming a set of nearly optimal models—an intriguing phenomenon known as the Rashomon effect in statistics.^30^^,^^31^^,^^53^ Within this Rashomon set of nearly optimal models, models can differ in their reliance on covariate information.^32^^,^^54^ This diversity leads to variations in fairness profiles, especially concerning sensitive variables, as displayed in Figures 2 and 3.

Let $f_{\left(U,S^{\prime }\right)}^{*}$ denote the optimal model that minimizes the expected loss in the model family $F_{\left(U,S^{\prime }\right)}$ that is built with less-sensitive variables $X_{U}$ and sensitive variables $X_{S^{\prime }}$. The $S^{\prime }$-specific Rashomon set is defined as

$$R_{\left(U,S^{\prime }\right)}\left(\epsilon_{0},f_{\left(U,S^{\prime }\right)}^{*},F_{\left(U,S^{\prime }\right)}\right)=\left\{f\in F_{\left(U,S^{\prime }\right)}|E\left[L\left(f,Y\right)\right]\leq \left(1+\epsilon_{0}\right)E\left[L\left(f_{\left(U,S^{\prime }\right)}^{*},Y\right)\right]\right\}$$

where near optimality is controlled by the small factor $\epsilon_{0}>0$. Particularly, for parametric models that can be fully represented by their coefficients $\beta_{\left(U,{\;S}^{\prime }\right)}$, i.e., a transparent model (for example, the family of logistic regression), the Rashomon set can be converted into a set of coefficients:

$$R_{\left(U,S^{\prime }\right)}\left(\epsilon_{0},\beta_{\left(U,S^{\prime }\right)}^{*},{Β}_{\left(U,S^{\prime }\right)}\right)=\left\{{\beta \in Β}_{\left(U,S^{\prime }\right)}|E\left[L\left(f_{\beta },Y\right)\right]\leq \left(1+\epsilon_{0}\right)E\left[L\left(f_{\left(U,S^{\prime }\right)}^{*},Y\right)\right]\right\}\text{,}$$

where ${Β}_{\left(U,S^{\prime }\right)}$ is the coefficient space of models in $F_{\left(U,S^{\prime }\right)}$ and $\beta_{\left(U,S^{\prime }\right)}^{*}$ is the coefficients of the optimal model $f_{\left(U,S^{\prime }\right)}^{*}$. For practical usage, the anchor of the Rashomon set—the expected loss—can also be replaced by a performance metric M (such as the AUC value) evaluated on the validation set, leading to

$$R_{\left(U,S^{\prime }\right)}\left(\epsilon_{0},\beta_{\left(U,S^{\prime }\right)}^{*},{Β}_{\left(U,S^{\prime }\right)}\right)=\left\{{\beta \in Β}_{\left(U,S^{\prime }\right)}|M\left(f_{\beta },Y\right)\geq \left(1-\epsilon_{0}\right)M\left(f_{\left(U,S^{\prime }\right)}^{*},Y\right)\right\}\text{.}$$

To objectively evaluate the effects of sensitive variables on model performance and fairness, we consider different cases of variable selection for $X_{S^{\prime }}$. The cases include no exclusion (i.e., $X_{\left(U,S\right)}$, the baseline case), complete exclusion (i.e., $X_{U}$, under blindness), and all possible cases of partial exclusion. We defined the integral Rashomon set (IRS) with all the cases of $S^{\prime }$ considered that can be expressed as

$$R\left(\epsilon ,\beta_{\left(U,\cdot \right)}^{*},{Β}_{\left(U,\cdot \right)}\right)=\underset{\begin{array}{c} S^{\prime }\subseteq S\\ \beta_{\left(U,S^{\prime }\right)}^{*}\in R_{\left(U,S\right)}\left(\epsilon_{0},\beta_{\left(U,S\right)}^{*},{Β}_{\left(U,S\right)}\right) \end{array}}{⋃}R_{\left(U,S^{\prime }\right)}\left(\epsilon_{0},\beta_{\left(U,S^{\prime }\right)}^{*},{Β}_{\left(U,S^{\prime }\right)}\right)\text{.}$$

The case-specific near optimality determined by $\epsilon_{0}$ should be more stringent to guarantee the overall near optimality of the IRS determined by ϵ, i.e., $\epsilon >\;\epsilon_{0}>0$ (see more details in supplemental experimental procedures S1). In previous studies, ϵ is often set at 5%.^32^^,^^54^

We employ the method of rejection sampling^32^^,^^55^ to identify the nearly optimal models, that is, to generate random samples of the coefficient vector and reject those with corresponding expected loss (or performance) out of near optimality. Specifically, to fully represent the case-specific Rashomon set $R_{\left(U,S^{\prime }\right)}\left(\epsilon_{0},\beta_{\left(U,S^{\prime }\right)}^{*},{Β}_{\left(U,S^{\prime }\right)}\right)$, the i-th sample of the coefficient vector is generated from a multivariable normal distribution $N\left(\beta_{\left(U,S^{\prime }\right)}^{*},k_{i}\Sigma_{\left(U,S^{\prime }\right)}^{*}\right)$, where $\beta_{\left(U,S^{\prime }\right)}^{*}$ and $\Sigma_{U,S^{\prime }}^{*}$ are the coefficient vector and variance-covariance matrix of the optimal model $f_{\left(U,S^{\prime }\right)}^{*}$ based on less-sensitive variables $X_{U}$ and sensitive variables $X_{S^{\prime }}$, respectively. Sampling guided by the characteristics of the optimal model enables the efficient generation of nearly optimal models and addresses model diversity within near optimality of performance. This approach can generate models with varied coefficients, as shown in Figure 2, leading to various fairness profiles while achieving nearly optimal performance as required by the reject sampling, which allows for the identification of fairer models. In addition, the control parameter of scope-width $k_{i}$ is drawn from a uniform distribution $U\left(u_{1},u_{2}\right)$ with tunable parameters $u_{1}$ and $u_{2}$ to adjust the scope of sampling.

### Model selection with contextualized fairness

To comprehensively rank the nearly optimal models in the IRS, i.e., $R\left(\epsilon ,\beta_{\left(U,\cdot \right)}^{*},{Β}_{\left(U,\cdot \right)}\right)$, and select the fairer one, we developed the FRI. Inspired by the radar chart for comparing items across multiple dimensions, the FRI is a holistic ranking measure that considers not only individual dimensions of fairness metrics ($m_{1},m_{2},\ldots ,m_{J}$) but also their interdependencies, calculated as

$$FRI\left(f_{\beta }\right)=\frac{1}{\sum_{j=1}^{J}m_{j}\left(f_{\beta }\right)m_{j+1}\left(f_{\beta }\right)}\text{,}$$

where $m_{J+1}∶=m_{1}$ to simplify notations. The highest-ranked model within $R\left(\epsilon ,\beta_{\left(U,\cdot \right)}^{*},{Β}_{\left(U,\cdot \right)}\right)$ is chosen as the default fairness-aware model:

$$\tilde{\beta }=\underset{\beta \in R\left(\epsilon ,\beta_{\left(U,\cdot \right)}^{*},{Β}_{\left(U,\cdot \right)}\right)}{\mathrm{argmax}}FRI\left(f_{\beta }\right)\text{,}$$

with a corresponding subset of sensitive variable(s) $X_{\tilde{S}}$. The FAIM framework prioritizes the fairness notion of ensuring the equality of model discriminatory performance across subgroups (also named “separation-based” fairness), focusing on making the prediction $\hat{Y}$ independent of sensitive variables $X_{S}$ when conditioned on the true outcome Y.^14^^,^^22^^,^^25^^,^^56^^,^^57^ This fairness notion encompasses various fairness metrics that essentially measure the gaps in performance (e.g., accuracy, AUC, sensitivity, specificity, etc.) among subgroups, with smaller gaps indicating better fairness.^56^ For example, equal opportunity^22^ emphasizes equal sensitivity (i.e., true positive rate) across subgroups, defined as the maximal discrepancy in sensitivity values across subgroups. Particularly, we used commonly used statistical metrics, including equalized odds,^22^ equal opportunity,^22^ and BER equality,^14^ as default options to compose the FRI (see more details in Table 1).

The process of model selection is designed to be interpretable and interactive. To facilitate such human-involved investigation, FAIM provides an interactive graphical interface (https://github.com/nliulab/FAIM) to display detailed information on each model upon hovering. As illustrated in Figure 3, which is based on SGH-ED data, Figure 3A visualized individual fairness metrics in each panel for the sampled nearly optimal models, with joint fairness ranked by the FRI. The top 1 model highlighted in gold is the default choice for the fairness-aware model. Nevertheless, users can assess alternative models with high rankings, such as the top 10 models, through a clinical lens, based on detailed data-driven evidence provided in Figures 3B and 3C. These high-ranked models may differ in how they handle sensitive variables yet have comparable model performance and fairness status. Clinical justification can jump in to support the final exclusion of sensitive variables. Therefore, the final fairness-aware model yielded by FAIM integrates domain knowledge with data-driven evidence to enhance model fairness without impairing performance.

As depicted in Figure 1, we investigate how variable importance shifts before and after fairness enhancement via variable importance analysis with SHAP. While we can directly compare the coefficients of the two logistic regression models, the value of coefficients is often not directly comparable between continuous and categorical variables.^58^ Therefore, we used SHAP as an alternative measure of variable importance to facilitate comparison between the fairness-unaware and fairness-aware models. The fairness-aware model $f_{\tilde{\beta }}$ shares a similar model architecture with the fairness-unaware $f_{\beta_{\left(U,S\right)}^{*}}$, except it may exclude certain sensitive variables that do not contribute to model performance and degraded fairness. The vector $\tilde{\beta }$ serves as a nearly optimal solution that minimally deviates from $\beta_{\left(U,S\right)}^{*}$ to execute fairness adjustments. In addition, $f_{\tilde{\beta }}$ holds the same functionality as $f_{\left(U,S\right)}^{*}$ in implementation. Take logistic regression for example: the coefficient $\tilde{\beta }$ can still be interpreted as the vector of log values of odds ratios, corresponding to the model $f_{\tilde{\beta }}=\frac{1}{1+e^{-\tilde{\beta }X_{\left(U,\tilde{S}\right)}}}$ and logit loss $L\left(f_{\tilde{\beta }},Y\right)\text{.}$ The standard errors for $\tilde{\beta }$ are estimated using Fisher’s information $I\left(\tilde{\beta }\right)$, mirroring the method used for $\beta_{\left(U,S\right)}^{*}$ based on $I(\beta_{\left(U,S\right)}^{*}$).

### Data and study design

To illustrate the clinical application of the FAIM framework, we used two datasets from different populations. The first dataset was derived from the MIMIC-IV-ED,^33^ a publicly available database of ED admissions at the Beth Israel Deaconess Medical Center (BIDMC) in the US between 2011 and 2019. The second dataset, referred to as the SGH-ED database,^34^ was acquired from a tertiary hospital in Singapore, with data of approximately 1.8 million ED visits between 2008 and 2020.

Our predictive models for hospital admission (Y), informed by a previous study,^59^ utilized predictors including demographic data, vital signs, triage score, and health records (see more details in Table S4). For MIMIC-IV-ED data, we excluded patients with an age below 18,^59^ while for SGH-ED data, we excluded patients who were not Singapore residents or were aged below 21.^54^ In MIMIC-IV-ED data, we recategorized race into five groups—Asian, Black, Hispanic, White, and others—and binarized the ESI into low risk (3–5) and high risk (1–2).^60^ In SGH-ED data, we recategorized race into four groups, Chinese, Indian, Malay, and others, and recategorized the triage score—PACS—into three levels: P1, P2, and P3–4.^61^ Given the interplay of racism and sexism,^23^^,^^24^ we treated race and sex as sensitive variables in predicting hospital admissions.

Each dataset was randomly split into training (70%), validation (10%), and test (20%) sets. We utilized the training set for the generation of nearly optimal models, the validation set for model selection with contextualized fairness, and the test set for model evaluation. In addition, to illustrate variable importance using SHAP, we utilized subsamples of training and validation sets, respectively, as background and explanation data.

### Statistical analysis

To compare the effects on fairness improvement from the baseline model, we included the well-validated pre-process method reweighing,^16^ the in-process methods reductions^19^ and Fair-GLM^62^ (regularization-based methods), and the post-process method equalized-odds post-processing^22^ for comparison. These methods of manipulating logistic regression to achieve fairness share a similar level of transparency with respect to the model setting. Additionally, we also included Adnet,^25^ a recent bias mitigation method that utilizes black-box models, particularly adversarial learning, to compare fairness achievement. See supplemental experimental procedures S2 for more implementation details. A conventionally trained logistic regression model served as the fairness-unaware baseline model. We evaluated model fairness with equalized odds, equal opportunity, and BER equality. We assessed model performance using the AUC, sensitivity, and specificity values, with 95% CIs reported. The sensitivity and specificity values were yielded based on optimal thresholds determined by Youden’s J statistics.^63^ The performance of two bias mitigation methods was deemed comparable if their AUC CIs overlapped. The data analysis and model building were performed using R v.4.0.2 (The R Foundation for Statistical Computing) and Python v.3.9.7.

## Resource availability

### Lead contact

Further information and requests for resources should be directed to and will be fulfilled by the lead contact, Nan Liu (liu.nan@duke-nus.edu.sg).

### Materials availability

This research did not generate any materials.

### Data and code availability

The MIMIC-IV-ED data are publicly available subject to the completion of ethics training and a signed data use agreement and are for research only. Due to ethical reasons and institutional guidelines, the SGH-ED data cannot be shared publicly. Data are available to researchers with some access restrictions applied upon request. Interested researchers may contact the SingHealth Health Services Research Center (hsr@singhealth.com.sg) for more details. The original codes have been deposited at Zenodo^64^ and are publicly available at GitHub, https://github.com/nliulab/FAIM.

## Acknowledgments

We thank Prof. Bin Yu and Mr. Zachary Thomas Rewolinski for their suggestions on improving this paper. This work was supported by the Duke-NUS Signature Research Programme funded by the 10.13039/501100001350Ministry of Health, Singapore. Any opinions, findings, and conclusions or recommendations expressed in this material are those of the authors and do not reflect the views of the Ministry of Health. Y.N. was supported by the Khoo Postdoctoral Fellowship Award (project no. Duke-NUS-KPFA/2021/0051) from the Estate of Tan Sri Khoo Teck Puat.

## Author contributions

M.L. and N.L. conceived and designed the study. M.L. and Y.N. wrote the code. M.L. performed the analyses and wrote the manuscript. M.L., Y.N., and N.L. made critical revisions to the manuscript. All authors interpreted the content, made revisions to the manuscript, and had final approval of the completed version. N.L. oversaw the project.

## Declaration of interests

The authors declare no competing interests.

## Declaration of generative AI and AI-assisted technologies in the writing process

During the preparation of this work, the authors used ChatGPT4.0 to improve the readability and language of the manuscript. After using this tool/service, the authors reviewed and edited the content as needed and take full responsibility for the content of the published article.

## Supplemental information

Document S1. Figures S1–S4, Tables S1–S6, Note S1, and supplemental experimental procedures

Document S2. Article plus supplemental information
