# Supplementary material for: FAIM: Fairness-aware interpretable modeling for trustworthy machine learning in healthcare
Source: Patterns (N Y). 2024 Sep 12;5(10):101059. doi: 10.1016/j.patter.2024.101059 (PMC11573921; doi:10.1016/j.patter.2024.101059)
Supplement: Document S1. Figures S1–S4, Tables S1–S6, Note S1, and supplemental experimental procedures [file mmc1.pdf]

Patterns, Volume *5*

Supplemental information

FAIM: Fairness-aware interpretable modeling for trustworthy machine learning in healthcare

Mingxuan Liu, Yilin Ning, Yuhe Ke, Yuqing Shang, Bibhas Chakraborty, Marcus Eng Hock Ong, Roger Vaughan, and Nan Liu

Supplemental information

Document S1. Figures S1-S2, Table S1-S4, Note S1, and Supplemental experimental procedures S1-2.

Figure S1. Distribution of model performance and coefficient variability for nearly optimal models (n=800) on MIMIC-IV-ED data.

A.

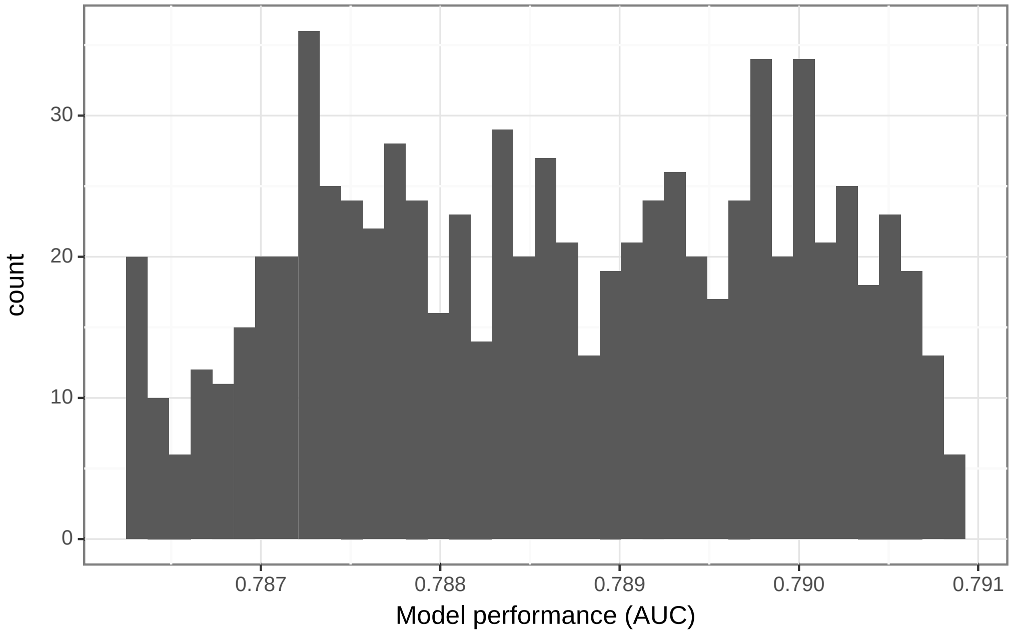

B.

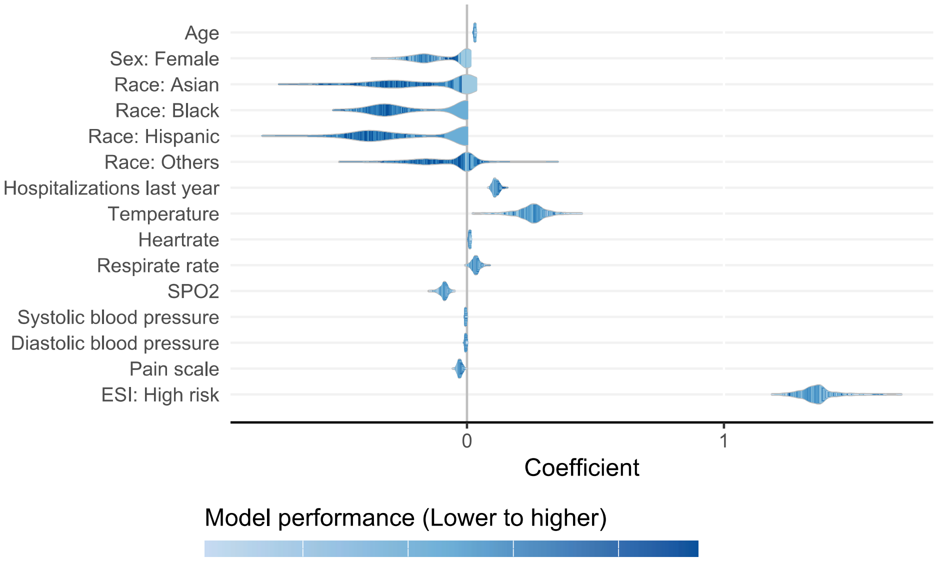

A. Histogram showing the distribution of model performance (AUC) for nearly optimal models, evaluated on the validation set. **B.** Violin plot depicting the variability of model coefficients across different variables. The width of each violin represents the range of coefficient values, while the color gradient indicates the level of model performance, with darker shades corresponding to higher performance. Variables of ESI risk, temperature, race, and sex display wide spectrums of coefficients.

Figure S2. The interactive plot for model selection, based on the nearly-optimal models' fairness evaluated on the validation set (MIMIC-IV-ED).

A.

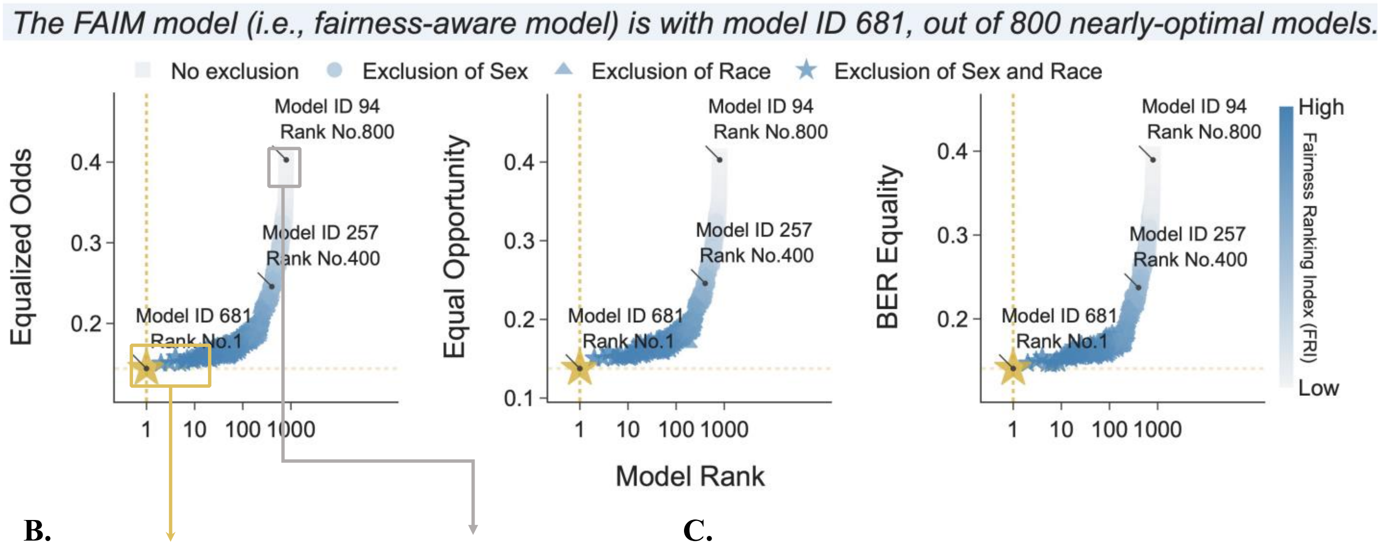

B.

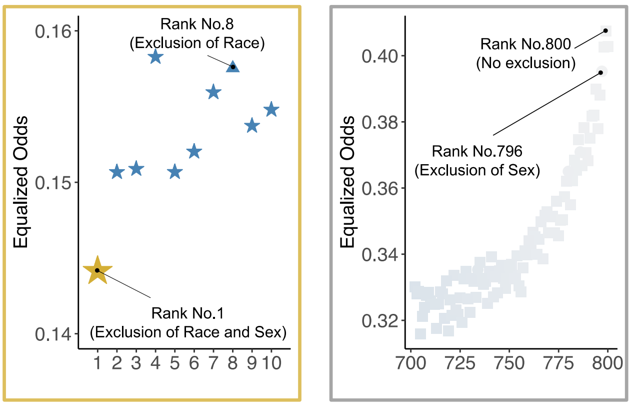

C.

Exclusion of Sensitive Variables on Fairness Rankings

Exclusion cases	1-10 ("most fair")	11-700	701-800 ("least fair")	Highest ranking
No exclusion	0	105	96	No.426
Exclusion of Race	1	199	0	No.8
Exclusion of Sex	0	196	4	No.284
Exclusion of Race and Sex	9	190	0	No.1

A. The graphical interface ranks nearly-optimal models based on fairness metrics—equalized odds, equal opportunity, and BER equality. Model 681 is ranked first by the Fairness Ranking Index (FRI), highlighted as the default fairness-aware model. Users can interactively engage with the data by hovering over points to display model details, zooming in on sections like the top-10 models, and adjusting panel views. **B.** Illustration of the panel of "equalized odds" metric with top-10 models showcased in the gold box, showing nine models (stars) excluding both sex and race, and one models (upwards triangle) excluding only race. The bottom-100 models, showcased in the grey box, predominantly include no exclusions (rectangles), with a minority excluding sex (upward triangles). **C.** A tabulation of models based on the exclusion cases of sensitive variable(s), detailing the counts of models in different fairness ranges—from "most fair" to "least fair". The right-most column records the highest ranking obtained by the nearly-optimal models for each exclusion case.

Table S1. Characteristics of MIMIC-IV-ED dataset

	Overall	Not admitted	Admitted	p-value
n	418100	220276	197824	
**Age, mean (SD)**	52.8 (20.6)	46.3 (19.4)	60.1 (19.5)	<0.001
**Sex, n (%)**				<0.001
Female	227007 (54.3)	126755 (57.5)	100252 (50.7)	
Male	191093 (45.7)	93521 (42.5)	97572 (49.3)	
**Race, n (%)**				<0.001
Asian	18321 (4.4)	11197 (5.1)	7124 (3.6)	
Black	92168 (22.0)	55944 (25.4)	36224 (18.3)	
Hispanic	34150 (8.2)	22158 (10.1)	11992 (6.1)	
White	242666 (58.0)	113445 (51.5)	129221 (65.3)	
Others	30795 (7.4)	17532 (8.0)	13263 (6.7)	
**ESI, n (%)**				<0.001
High risk[1-2]	163430 (39.1)	48103 (21.8)	115327 (58.3)	
Low risk[3-5]	254670 (60.9)	172173 (78.2)	82497 (41.7)	
**Systolic blood pressure, mean (SD)**	134.9 (22.2)	135.2 (20.7)	134.5 (23.7)	<0.001
**Heartrate, mean (SD)**	85.0 (17.4)	83.9 (16.3)	86.3 (18.6)	<0.001
**Diastolic blood pressure, mean (SD)**	77.5 (14.7)	78.8 (13.8)	76.0 (15.6)	<0.001
**Temperature, mean (SD)**	36.7 (0.5)	36.7 (0.5)	36.7 (0.6)	<0.001
**Pain scale, mean (SD)**	4.2 (3.6)	4.7 (3.6)	3.6 (3.5)	<0.001
**SpO_2, mean (SD)**	98.4 (2.4)	98.8 (2.0)	97.9 (2.7)	<0.001
**Respiratory rate, mean (SD)**	17.6 (2.5)	17.3 (2.1)	17.9 (2.8)	<0.001
**Hospitalizations last year, mean (SD)**	1.0 (2.7)	0.6 (2.2)	1.4 (3.1)	<0.001

ESI: Emergency Severity Index; SpO_2: oxygen saturation as detected by the pulse oximeter.

Table S2. Characteristics of SGH-ED dataset

	Overall	Not admitted	Admitted	p-value
n	1716830	1074513	642317	
**Age, mean (SD)**	53.1 (19.3)	47.3 (18.0)	62.6 (17.4)	<0.001
**Sex, median [Q1,Q3]**				<0.001
Female	822284 (47.9)	511060 (47.6)	311224 (48.5)	
Male	894546 (52.1)	563453 (52.4)	331093 (51.5)	
**Race, median [Q1,Q3]**				<0.001
Chinese	1114566 (64.9)	660744 (61.5)	453822 (70.7)	
Indian	229058 (13.3)	158004 (14.7)	71054 (11.1)	
Malay	195334 (11.4)	121211 (11.3)	74123 (11.5)	
Other Races	177872 (10.4)	134554 (12.5)	43318 (6.7)	
**PACS, n (%)**				<0.001
P1	202617 (11.8)	54231 (5.0)	148386 (23.1)	
P2	730445 (42.5)	365453 (34.0)	364992 (56.8)	
P3_P4	783768 (45.7)	654829 (60.9)	128939 (20.1)	
**Systolic blood pressure, mean (SD)**	132.1 (22.9)	131.2 (21.2)	133.6 (25.5)	<0.001
**Pulse, mean (SD)**	81.4 (15.8)	80.7 (14.4)	82.6 (17.8)	<0.001
**Diastolic blood pressure, mean (SD)**	73.1 (12.8)	74.0 (11.9)	71.6 (14.1)	<0.001
**Temperature, mean (SD)**	36.6 (0.6)	36.5 (0.6)	36.6 (0.6)	<0.001
**SaO_2, mean (SD)**	98.2 (3.2)	98.4 (3.0)	97.9 (3.6)	<0.001
**Respiratory rate, mean (SD)**	17.6 (1.5)	17.4 (1.3)	17.8 (1.8)	<0.001
**ED visits last year, mean (SD)**	1.3 (4.8)	1.1 (5.4)	1.5 (3.5)	<0.001

PACS: Patient Acuity Category Scale; SaO_2: oxygen saturation of arterial blood.

Table S3. Computation time of model training and testing for bias-mitigation methods on MIMIC-IV-ED data and SGH-ED data[1]

	MIMIC-IV-ED data (n = 418,100)[2]	SGH-ED data (n = 1,716,830)[2]	COMPAS data (n = 6,172)
Baseline	<1 min	<1 min	<1 min
Under blindness	<1 min	<1 min	<1 min
Reweigh	<1 min	<1 min	<1 min
Reductions	~0.5h	~0.5h	<1 min
Equalized odds post-processing	<1 min	<1 min	<1 min
Fair-GLM[3]	~1h	~1h	~5min
Adnet[4]	>30h for grid-search tuning + ~30min for retrainig	>30h for grid-search tuning + ~30min for retrainig	>30h for grid-search tuning +~5h for retrainig
FAIM[5]	~30min for metric-based or ~5min for loss-based Rashomon set + 15min model explanation	~30min for metric-based or ~5min for loss-based Rashomon set + 15min model explanation	~5min for metric-based or <1min for loss-based Rashomon set + ~10min model explanation

[1] The computations were performed on an HP Z6 G4 workstation equipped with an Intel Xeon Silver 4210 CPU (20 cores, 2.20 GHz), 128GB RAM, and an NVIDIA Quadro RTX 4000 GPU.
[2] The total sample size including training, validation, and test sets
[3] Given the computational capacity required, we sampled the original training set with stratification to sex, race, and the outcome of interest. The sampling ratios were 1/10 for MIMIC-ED data and 1/20 for SGH-ED data, which were consistent with the maximum dataset mentioned in the original Fair-GLM paper.
[4] The training becomes more unstable when the less-sensitive variables are embedded with obvious systematic biases.
[5] The metric-version of the Rashomon set in FAIM, requiring fairness evaluation of nearly-optimal models on the validation set, will take additional time. Computation time scales linearly with sample size and the number of sensitive variables.

Table S4. Variable selection

	MIMIC-IV-ED data	SGH-ED data
Demographic data	age, race, sex	age, race, sex
Vitals	temperature, bp-systolic, bp-diastolic, heartrate, respirate rate, pain	temperature, bp-systolic, bp-diastolic, pulse, respirate rate
Triage score	ESI	PACS
Oxygenation	SpO_2	SaO_2
Health record	no. hospitalization in one year	no. emergency visit in one year

ESI: Emergency Severity Index; PACS: Patient Acuity Category Scale; SpO_2: oxygen saturation as detected by the pulse oximeter; SaO_2: oxygen saturation of arterial blood.

Note S1. Summarized results of FAIM on COMPAS dataset with the prediction of two-year-recidivism, presented with Table S5-6 and Figure S3-4.

The predictors included age, number of priors, misdemeanor, days in jail, days in custody, number of juvenile felonies, number of juvenile misdemeanors, number of juvenile other offenses, charge degree. Gender and race were considered sensitive variables, but did not serve as predictors.[1]

Table S5. Two-dimension evaluation of model fairness and performance for bias mitigation methods using COMPAS data.

	Fairness metrics[1]			Performance metrics		
	Equal Opportunity	Equalized Odds	BER equality[2]	AUC	Sensitivity[3]	Specificity[3]
Baseline[4]	0.587	0.587	0.507	0.720 [0.692, 0.749]	0.619 [0.578, 0.660]	0.721 [0.686, 0.753]
Under blindness[5]	0.349	0.349	0.298	0.717 [0.688, 0.745]	0.676 [0.637, 0.717]	0.651 [0.615, 0.688]
FAIM	0.263	0.263	0.306	0.702 [0.673, 0.731]	0.527 [0.717, 0.721]	0.770 [0.738, 0.802]
Reweigh (pre-process)	0.349	0.349	0.298	0.716 [0.687, 0.744]	0.676 [0.637, 0.714]	0.651 [0.614, 0.688]
Reductions (in-process)	0.120	0.120	0.097	0.517 [0.495, 0.537]	0.187 [0.157, 0.219]	0.845 [0.817, 0.872]
Fair-GLM (in-process)	0.069	0.113	0.091	0.507 [0.475, 0.539]	0.904 [0.879, 0.929]	0.135 [0.110, 0.160]
Equalized odds post-processing (post-process)	0.615	0.615	0.499	0.522 [0.500, 0.545]	0.242 [0.208, 0.279]	0.802 [0.772, 0.832]
Adnet[6] (in-process)	0.290	0.290	0.264	0.708 [0.679, 0.737]	0.609 [0.568, 0.649]	0.719 [0.685, 0.753]

[1] Smaller values indicate higher levels of fairness
[2] BER equality: equality of the combination of true positive rate and true negative rate
[3] The thresholds were determined by Youden's J index for methods that can yield predictive probabilities (i.e., original logistics regression, "FAIM", "Reweigh", "Under blindness", "Fair-GLM", "Adnet"). In-process method "Reductions" and post-process method "equalized-odds post-process" directly generated the binary prediction.
[4] Baseline: the original logistics regression model, i.e., fairness-unaware model, with gender and race included. All of the other models do not include race and gender in modeling because it is unethical that the prediction of 2-year-recidcism is based on gender or race[2]. In FAIM, the default model did not exclude race. However, we chose the highest-ranking model that excluded both race and gender as the output model.
[5] Under blindness: the logistics regression with sensitive variables excluded
[6] Adnet: bias-mitigation method utilizing adversarial learning, i.e., black-box models, instead of manipulating regression models to achieve fairness.

The results demonstrate the benefits of FAIM in improving fairness while maintaining performance. Additionally, the findings also suggest that many bias-mitigation methods may lead to extreme sensitivity or specificity values to achieve fairness, potentially causing significant degradation in model performance, both overall and subgroup-wise performance.

Table S6. Subgroup analysis regarding gender and race (method yielding highlighted in bold)

		Baseline[1]	Under blindness	FAIM
Race	ΔTPR[2]	0.268	0.210	**0.185**
	ΔTNR[3]	0.254	0.220	**0.171**
Gender	ΔTPR	0.399	0.169	**0.167**
	ΔTNR	0.208	**0.041**	0.079

[1] Baseline: the original logistics regression model, i.e., fairness-unaware model, with gender and race included.
[2] ΔTPR: the gap of true positive rate among race/ethnicity or gender subgroups
[3] ΔTNR: the gap of true negative rate among race/ethnicity or gender subgroups

Figure S3. Variable importance analysis in both fairness-unaware model (i.e., "Baseline") and fairness-aware (i.e, "FAIM") model based on COMPAS datasets.

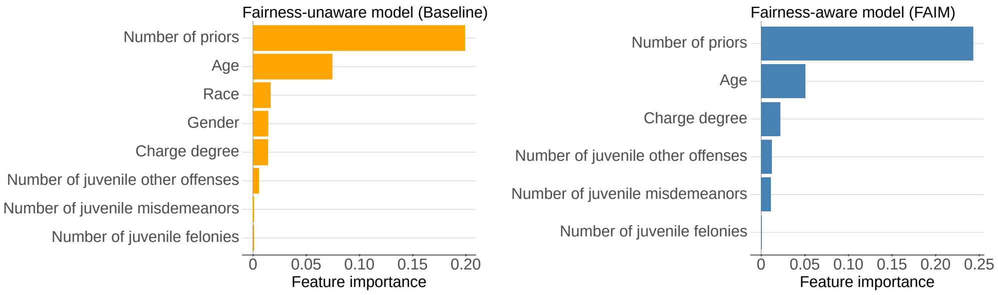

The fairness-aware model refers to the ranking No.1 fairest model yielded by FAIM, and the fairness-unaware model refers to the baseline model, i.e. the logistics regression model.

Figure S4. Comparison of the odds ratios between fairness-unaware ("Baseline") and fairness-aware ("FAIM") models based on COMPAS data.

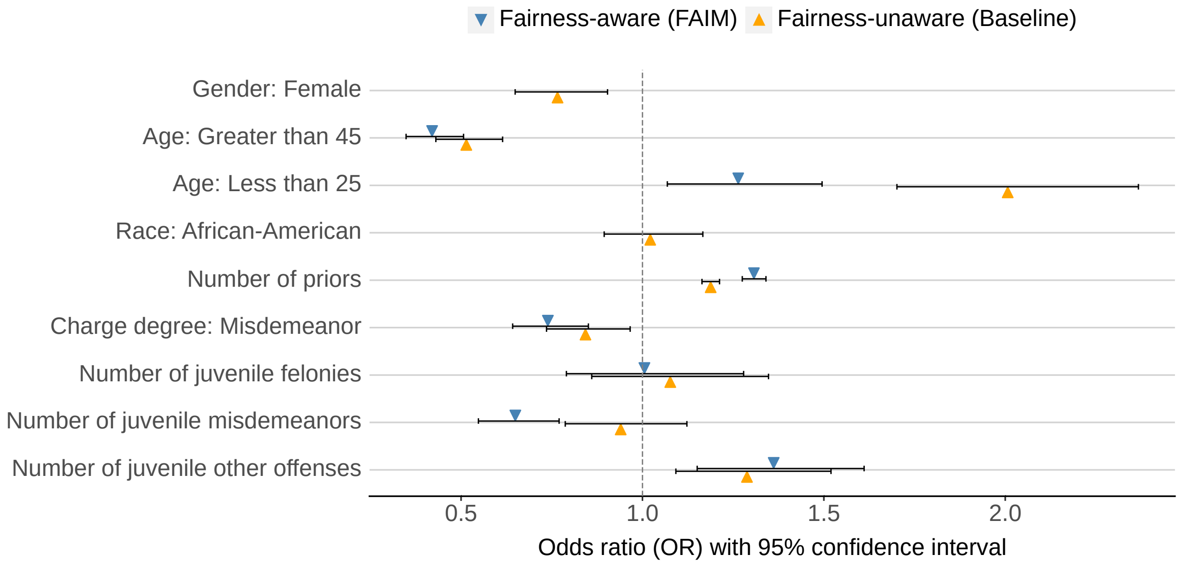

Supplemental experimental procedures

S1. Mechanism of ϵ and ϵ_0 for defining Rashomon set

The Integral Rashomon Set (IRS), $R(\epsilon, \beta^*_{(U,\cdot)}, \mathrm{B}_{(U,\cdot)})$, represents a "double near-optimal" subset of the actual Rashomon set, $R(\epsilon, \beta^*_{(U,S)}, \mathrm{B}_{(U,S)})$. In other words, the exclusion case of feature subset S/S' will be considered and the S'-specific cloud of "nearly optimal" models, $R_{(U,S')}\left(\epsilon_0, \beta^*_{(U,S')}, \mathrm{B}_{(U,S')}\right)$, will contribute to the composition of IRS, if and only if the S'-specific optimal model, $\beta^*_{(U,S')}$, is "nearly optimal" compared to the optimal modal, $\beta^*_{(U,S)}$, obtained in the original case:

For the loss version, if $\beta \in R(\epsilon, \beta^*_{(U,\cdot)}, \mathrm{B}_{(U,\cdot)})$, then there exists a subset $S' \subseteq S$ such that $E[L(f_\beta, Y)] \leq (1+\epsilon_0)\mathrm{E}\left[L\left(f^*_{(U,S')}, Y\right)\right]$ where $f^*_{(U,S')}$ is equivalent to $f_{\beta^*_{(U,S')}}$. Since $\beta^*_{(U,S')} \in R_{(U,S)}(\epsilon_0, \beta^*_{(U,S)}, \mathrm{B}_{(U,S)})$ and $\epsilon = (\epsilon_0 + 1)^2 - 1$, we have $\mathrm{E}\left[L\left(f^*_{(U,S')}, Y\right)\right] \leq (1+\epsilon_0)\mathrm{E}[L(f^*_{(U,S)}, Y)]$. Thus,

$$\begin{aligned} E[L(f_\beta, Y)] &\leq (1+\epsilon_0)\mathrm{E}\left[L\left(f^*_{(U,S')}, Y\right)\right] \\ &\leq (1+\epsilon_0)^2\,\mathrm{E}[L(f^*_{(U,S)}, Y)] \\ &= (1+\epsilon)\mathrm{E}[L(f^*_{(U,S)}, Y)]. \end{aligned}$$

That is, $\beta \in \mathrm{R}(\epsilon, \beta^*_{(U,S)}, \mathrm{B}_{(U,S)})$.

Similarly, for the metric M version (e.g., AUC), if $\beta \in R(\epsilon, \beta^*_{(U,\cdot)}, \mathrm{B}_{(U,\cdot)})$, then there exists a subset $S' \subseteq S$ such that $M(f_\beta, Y) \geq (1-\epsilon_0)M\left(f^*_{(U,S')}, Y\right)$ where $f^*_{(U,S')}$ is equivalent to $f_{\beta^*_{(U,S')}}$. Since $\beta^*_{(U,S')} \in R_{(U,S)}(\epsilon_0, \beta^*_{(U,S)}, \mathrm{B}_{(U,S)})$ and $\epsilon = 1 - (\epsilon_0 - 1)^2$, we have $M\left(f^*_{(U,S')}, Y\right) \geq (1-\epsilon_0)M(f^*_{(U,S)}, Y)$. Thus,

$$\begin{aligned} M(f_\beta, Y) &\geq (1-\epsilon_0)M\left(f^*_{(U,S')}, Y\right) \\ &\geq (1-\epsilon_0)^2 M(f^*_{(U,S)}, Y) \\ &\geq (1-\epsilon)M(f^*_{(U,S)}, Y). \end{aligned}$$

That is, $\beta \in \mathrm{R}(\epsilon, \beta^*_{(U,S)}, \mathrm{B}_{(U,S)})$.

S2. Implementation setting details

The implementation of commonly used methods Reweigh, Reductions, and Equalized odds post-processing was based on the Python libraries "AIF360" and "Fairlearn". To match the transparency in model setting with the baseline fairness-unaware model, we implemented Reduction method based on logistic regression. In addition, we incorporated a recent method "Fair-GLM" and implemented it based on the codes available in its GitHub repository (https://github.com/hyungrok-do/fair-glm-cvx). Given the computational capacity required, we sampled the original training set with stratification to sex, race, and the outcome of interest. The sampling ratios were 1/10 for MIMIC-ED data and 1/20 for SGH-ED data, which were consistent with the maximum dataset mentioned in the original Fair-GLM paper. We implemented "Adnet" with respectively two three-layer multilayer perceptron with LeakyReLU activations as predictor and discriminator. Adam optimizer was used with a StepLR scheduler to set the learning rates. The learning rates were reduced by a factor of 0.99 every 10 steps. If there was no improvement in the monitored metric (AUC) for 10 consecutive epochs, the training process is stopped early. The grid-search for hyperparameter tuning is as below, with extreme hyperparameter values tested in pivot training:

- Initial learning rate for predictor: 1e-5, 1e-4, 1e-3
- Initial learning rate for discriminator: 1e-5, 1e-4, 1e-3
- Coefficient for adversarial: 0.1, 0.5, 1

Supplemental references

1. Fisher, A., Rudin, C., and Dominici, F. (2019). All Models are Wrong, but Many are Useful: Learning a Variable's Importance by Studying an Entire Class of Prediction Models Simultaneously. Journal of Machine Learning Research *20*, 1–81-81–81.
2. Scurich, N., and Monahan, J. (2016). Evidence-based sentencing: Public openness and opposition to using gender, age, and race as risk factors for recidivism. Law and Human Behavior *40*, 36-41. 10.1037/lhb0000161.
